# Clinical and Laboratory Approach to Diagnose COVID-19 Using Machine Learning

**DOI:** 10.1007/s12539-021-00499-4

**Published:** 2022-02-08

**Authors:** Krishnaraj Chadaga, Chinmay Chakraborty, Srikanth Prabhu, Shashikiran Umakanth, Vivekananda Bhat, Niranjana Sampathila

**Affiliations:** 1 Department of Computer Science and Engineering, Manipal Institute of Technology, Manipal Academy of Higher Education, Manipal, India; 2 Department of Electronics and Communication, Birla Institute of Technology, Mesra, India; 3 Department of Medicine, Dr. TMA Hospital, Manipal Academy of Higher Education, Manipal, India; 4 Department of Biomedical Engineering, Manipal Institute of Technology, Manipal Academy of Higher Education, Manipal, India

**Keywords:** Artificial Intelligence, Machine Learning, COVID-19, Blood tests, RT-PCR

## Abstract

Coronavirus 2 (SARS-CoV-2), often known by the name COVID-19, is a type of acute respiratory syndrome that has had a significant influence on both economy and health infrastructure worldwide. This novel virus is diagnosed utilising a conventional method known as the RT-PCR (Reverse Transcription Polymerase Chain Reaction) test. This approach, however, produces a lot of false-negative and erroneous outcomes. According to recent studies, COVID-19 can also be diagnosed using X-rays, CT scans, blood tests and cough sounds. In this article, we use blood tests and machine learning to predict the diagnosis of this deadly virus. We also present an extensive review of various existing machine-learning applications that diagnose COVID-19 from clinical and laboratory markers. Four different classifiers along with a technique called Synthetic Minority Oversampling Technique (SMOTE) were used for classification. Shapley Additive Explanations (SHAP) method was utilized to calculate the gravity of each feature and it was found that eosinophils, monocytes, leukocytes and platelets were the most critical blood parameters that distinguished COVID-19 infection for our dataset. These classifiers can be utilized in conjunction with RT-PCR tests to improve sensitivity and in emergency situations such as a pandemic outbreak that might happen due to new strains of the virus. The positive results indicate the prospective use of an automated framework that could help clinicians and medical personnel diagnose and screen patients.

**Graphical abstract:**

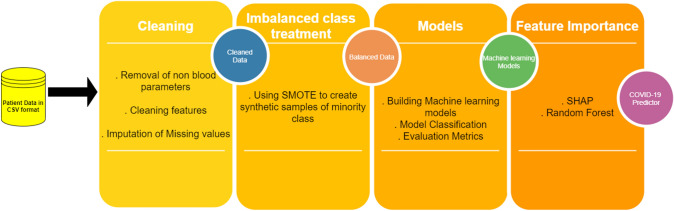

## Introduction

The Coronavirus is an extremely dangerous infection transmitted by the Severe Acute Respiratory Coronavirus 2 (SARS-CoV-2) that has rapidly spread around the world. It has turned out to be an extremely fatal disease and is the reason for more than 500,000 deaths across 216 countries [1]. Every aspect of human activity has been impacted severely in all geographic territories and quick detection and treatment of this virus is extremely crucial to avert the escalation of this infectious virus. Currently, COVID-19 is commonly diagnosed using RT-PCR (Reverse Transcription Polymerase Chain Reaction) along with the Rapid Antigen tests (RAT) [2]. These tests are time-consuming and about 20% false negative rates have been observed [3]. A large number of underdeveloped nations do not have accessibility to RT-PCR testing kits. RAT testing is based on IgM/IgG antibodies. Low specificity (77.8%) and sensitivity (18.8%) have been the main drawbacks of this method [4]. Therefore, emphasis is being given to other methods of testing that might be more accessible and less expensive in the future. One of the most trending concepts in the modern world is Artificial Intelligence (AI). Various aspects such as Machine Learning (ML), modelling, statistics, simulations and algorithms are included in the above concept. It also contributes significantly to clinical and academic research [5]. Engineering, medical, psychology, sociology, hazard mitigation, multi-disciplinary science and other fields can efficiently make use of ML in the future. Numerous applications of Machine Learning (ML) have been utilized in activities such as sanitizing places with drones [6], tracking users using face recognition, drug development, automated robots delivering medicine and food, COVID-19 diagnosis, etc. According to the current literature, ML and hybridised models have been successfully applied in several domains of engineering [7–10], psychometric analysis [11, 12], medical and pharmaceutics [13–15], graph theory [16], and social sciences [17–19].

A considerable interest has been taken by various researchers in examining the field of AI and ML applications in battling this deadly virus by effectively deploying them in forecasting, diagnosis and prognosis, drug discovery and disease surveillance [20, 21]. ML techniques have been deployed to help health care specialists with rapid, reliable and accurate detection of the novel coronavirus in this article. Computed Tomography Scans (CT-Scans) and chest X-ray images (XSR) images along with AI based medical imaging have been successfully used to detect the viral disease. Biomedical image analysis using AI has gained a lot of prominence and a lot of articles have been published with a sole focus on CT-Scans and X-rays [22–25]. However, the radiation doses emitted during CT-Scans can cause cancer. High cost and availability of CT-Scanners is also an issue. Research has also taken place in exploring the use of cough sounds for COVID-19 diagnosis using NLP (Natural Language Processing) [26–28].

The blood and laboratory markers of COVID-19 patients can change drastically and these parameters can be used in the preliminary screening according to a numerous number of medical studies [29–33]. The presence of this infection can be confirmed by diagnosis, while a probabilistic indication of the disease's presence can be provided by a round of initial screening tests. It is very difficult for a doctor/physician to extract complete information from different laboratory blood tests. But, various patterns obtained from blood parameters can be easily differentiated by the AI models. Therefore, development of ML models that can diagnose COVID-19 has been explored by many ML researchers and enthusiasts [34–36].

The ML framework using blood tests for COVID-19 detection can lead to an accessible, less expensive, easy to use and faster alternative to time-consuming and expensive tests. Furthermore, these tests can be utilised in conjunction with RT-PCR testing to avoid false negatives. Blood test-based tests can be used in poor and underdeveloped countries that suffer from a lack of technology and laboratory supplies. This inexpensive system can also speed up testing and maintain a smooth flow of patients [37, 38]. The main findings and contributions of this article are given below:

- An exhaustive review of various ML applications that diagnose COVID-19 using various blood and laboratory markers.
- An in-depth data analysis that reveals crucial and critical blood markers that are key in diagnosing coronavirus.
- Different machine-learning models that accurately detect COVID-19 from a variety of clinical indicators.
- Shapley Additive Explanations (SHAP) and random forest technique were used to validate feature importance. It was observed that platelets, leukocytes, monocytes and eosinophils were the most critical markers that may signify the occurrence of coronavirus for our data.
- Additional information about the various blood parameters that are critical in the diagnosis of the novel COVID-19 virus.

The aim and objective of this article is to introduce a ML based diagnosis framework that detects COVID-19 using routine blood parameters. Accuracy, recall, specificity, sensitivity, f1-score, AUC and brier score were the metrics used to evaluate our models to understand the advantages and disadvantages of the classifiers in this extensive study.

**Fig. 1:**
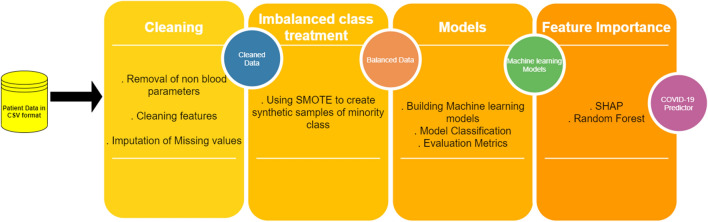
Integral learning steps required for the development of ML classifiers

The Random forest classifier achieved the best results in diagnosing COVID-19 for the dataset that was available publicly from Hospital Israelita Albert Einstein, Brazil. The Synthetic Minority Oversampling Technique (SMOTE) was employed to prevent imbalance in the data distribution since the dataset was extremely unbalanced. The Shapley Additive Explanations (SHAP) and the random forest approaches were utilized to calculate importance of the features and Pearson’s co-relation was used to find the various hidden co-relations between the various blood parameters and its relationship with this contagious virus. In this study, we first perform an extensive review of the existing literature in Sect. 2. Exploratory data analysis and the proposed design methodology are outlined in Sect. 3, followed by the evaluation of models in Sect. 4. Various challenges and the directions for future researches are described in Sect. 5. The article concludes in Sect. 6. Description of various techniques required for the design and development of the prospective ML models is given in Fig. 1.

## Related Work

This section consolidates a number of ML researches that have been used to diagnose COVID-19. These infections are increasing at a rapid rate and it is of utmost importance to identify patients early to avoid the large scale spread of the contagious disease. RT-PCR is the existing standard procedure for diagnosing coronavirus and samples are accumulated from the respiratory tracts. Further, PCR amplification is conducted after the RNA has been extracted successfully using a predefined medical protocol. This unique method is still the golden standard for diagnosis. However, it still has a number of limitations. Specialist equipment and trained personnel are required to execute this test [39]. Testing a single sample is not feasible since it is very expensive and can take a lot of time (4 to 5 h). To reduce costs, PCR machines are used with a number of samples. False negative rates have been found at a rate estimated to be between 3 and 30%. [40]. These incorrect results are dangerous since the patient will not be isolated and can cause further spread of the disease. CT-Scans have been used as an alternative to PCR tests [41, 42]. However, they cannot confirm the exact diagnosis of this viral disease. CT-Scans are not available everywhere and they can also cause exposure to unnecessary radiation [43]. Hence, doctors do not recommend CT-Scans and chest radiographs (CXR) for every single patient [44]. Clinical and routine blood tests can be used as an inexpensive and quick means of COVID-19 detection. These accurate algorithms can be used efficiently, especially during a pandemic peak when there is an acute shortage of hospital resources [45, 46]. Validation of RT-PCR tests maybe be conducted to reduce false negatives and increase the sensitivity using these blood test classifiers [47, 48]. Some researchers have used one particular model, others have chosen multiple models and some of the predictive models are a combination of many models. Various ML models that diagnose COVID-19 are described below. The rest of the articles, along with their key characteristics, are described in Table 1.

Wu et al. [49] presented the first model that diagnosed COVID-19 from routine blood parameters. They used 11 parameters out of the initial 49 parameters for training the ML model. A combination of 235 (105 COVID-19) patients were used in this research. The accuracy, specificity and sensitivity obtained were 95.95%, 95.13%, and 96% respectively for the external dataset. The blood parameters of 279 patients (177 COVID-19) from San Raffael Hospital were collected in the study [36]. Fourteen important blood parameters were given as input features to the various machine-learning classifiers. The accuracy obtained was 82–86% and the sensitivity obtained was 92–95%. The paper also concluded that AST (Aspartate Aminotransferase), lymphocytes, LDH (Lactate dehydrogenase), WBC (White Blood Cells) and CRP (C-Reactive protein) were the most important diagnostic blood parameters.

Kukar et al. [50] utilized ML models to predict the presence of coronavirus using the laboratory and clinical markers of 160 COVID-19 patients hospitalised in the University Medical Centre Ljubljana in Slovenia. The sample size also included 5333 COVID-19 negative patients. The classifiers utilised were Random Forest (RF), DNN (Deep Neural Network), and XGBoost (Extreme Gradient Boosting), with XGBoost producing the best results. The average sensitivity and AUC (Area Under Curve) achieved by the various methods were 88.9% and 97% respectively. Hypoalbuminemia (low levels of albumin) was observed in patients.

Fernandes et al. [51] investigated the blood laboratory markers of 235 COVID-19 patients admitted in Israelita Albert Einstein Hospital, Brazil. Fifteen distinct blood parameters were utilised as features, and the Support Vector Machine (SVM) produced the optimal prediction. The AUC, sensitivity, and specificity obtained were 85%, 68%, and 85%, respectively. According to the study, the most critical blood indicators were lymphocytes, leukocytes, and eosinophils. Alves et al. [34] used three ML algorithms to diagnose coronavirus from routine blood parameters. The sample consisted of 84 COVID-19 patients along with 608 other patients. The Local Decision Tree Explainer (DTX), criteria graphs and the random forest were the models used for classification. The random forest algorithm achieved optimal predictions with an accuracy, f1-score, sensitivity, specificity and AUROC of 88%, 76%, 66%, 96% and 86% respectively.

Plante et al. [52] used ML models to rule out SARS-CoV-2 using various clinical tests. 2183 PCR confirmed patients from 43 hospitals from the United States of America were included in this research. These models generate a risk score out of 10 (0 being minimal risk and 10 being maximum risk). The XGBoost model was the best performing model that achieved a sensitivity and an AUROC score of 95.9% and 91% respectively. Arpaci et al. [53] utilized 14 clinical characteristics to predict COVID-19 infection and the dataset included 114 confirmed COVID-19 cases from Taizhou hospital in China's Zhejiang province. Six distinct classifiers were employed and logistic regression produced the best results with 84.21% accuracy. Sobrinho et al. [54] used ML models to prioritize patients for testing based on various blood markers. The dataset consists of 55,676 patients along with 12 features. Eight different models were used for training/testing. Out of these, six models achieved high performance with the decision tree achieving the optimal results with an accuracy of 89.12%. LDH and CRP were the most distinctive features that could diagnose COVID-19, according to [55]. 15 features were used for the seven deployed ML models that were combined together. They achieved an AUROC and sensitivity of 91% and 93% respectively. However, the specificity obtained was poor (64%). In another study, Logistic regression (LR), neural network models and random forest (RF) were utilized in COVID-19 diagnosis [56]. Twenty-three clinical feature variables were utilised in the models described above. The dataset consisted of 536 (106 COVID-19) patients from Rennes-Academic Hospital, France. The LR model obtained the best results with an AUROC of 93%.

**Table 1:** List of ML models that diagnose COVID-19

References	Source	Size	Total attributes	Models used	Accuracy of best model	Sensitivity of best model	Specificity of best model	AUC of best model
[57]	Hospital Israelita Albert Einstein, Brazil	5644, 559 COVID-19	24 attributes	MLP (Multi-layer perceptron), SVM, DT, NB	95%	96%	93%	
[58]	Three Open access datasets	–	Many features	Machine learning and Deep Learning models		92%	82%	92%
[59]	18 hospitalls from Zhejiang, China	914 patients	10 features	LR, SVM,DT,RF,RL		95%	87%	97%
[60]	Tongji Hospital, China	413 patients	21- categorical, 21- continuous	Xgboost		92.5%	97.5%	
[61]	West China Hospital,m China	620 samples	9 features	Multi variate logistic regression	–	–	–	–
[62]	11 regions in China	659 patients	Many biochemical and clinical features	Decision trees	89%	–	–	88%
[63]	SMART hospitals	–	–	NB, RF, SVM	93.33%	–	–	–
[33]	Hospital Israelita Albert Einstein Hospital, Brazil	5644, 559 COVID-19 patients	Many blood parameters	ERLX, an ensemble learning model	99.60%	98.72%	98.99%	99.38%
[64]	UK Biobank	4510 patients	–	Linear discriminant analysis	–	–	–	97%
[65]	Hospital Israelita Albert Einstein Hospital, Brazil	5644 patients 598 COVID-19 patients	Many blood parameters	RF, Shallow learning, flexible ANN	–	–	–	95%
[66]	Hospital Israelita Albert Einstein Hospital, Brazil	5644 patients 598 COVID-19 patients	Many blood parameters	Er-CoV	–	70%	85%	86%
[67]	Kepler University Hospital	1357 patients	28 unique features	Random forest	86%	–	–	74%
[68]	Three Brazilian Hospitals	815 (442 COVID-19)	19 features	ADA boost, Gradient boosting, Random forest, extreme gradient boosting, SVM, partial least square	–	96%	93%	–
[69]	–	1521 patients	130 clinical features	HUST-19 (CNN based framework)	94%	–	–	–
[70]	Oxford University hospitals	1,14,957—negative 437—COVID-19	–	Various ML classifiers		77%	95%	93%
[71]	Five hospitals in New York	4098 COVID-19 patients	Many blood parameters	XGBoost	–	–	–	89%
[72]	–	279 cases	13 features	KNN, DT, RF, SVM, RF	–	–	–	91%

## Materials and Methods

### Dataset Description

The case data for this research was procured from 5644 patients who were hospitalized in the Albert Einstein Israelita Hospital, Brazil. To totally anonymize the data, best practises and standards were employed. The clinical data had already been standardised to get the best normal distribution possible (standard deviation = 1, mean = 0). This dataset was made available publicly and is often updated for collaborative research [73]. It includes the blood test reports of all in-patients who have been tested for Sars-CoV-2 virus (both positive and negative). 111 features that include various urine, blood and other medical tests of 5644 patients are included in this publicly available data. However, the dataset is extremely unbalanced, with very few positive cases (558) compared to a large number of negative cases. The various blood parameters include haemoglobin, haematocrit, platelets, red blood cells, leukocytes, lymphocytes, basophils and many more. Urine tests and tests for other contagious diseases were also included. RT-PCR results were used to confirm the patients’ diagnosis and were represented as dichotomous ground truth values (positive/negative).

### Data Pre-processing and Co-relation Analysis

**Fig. 2:**
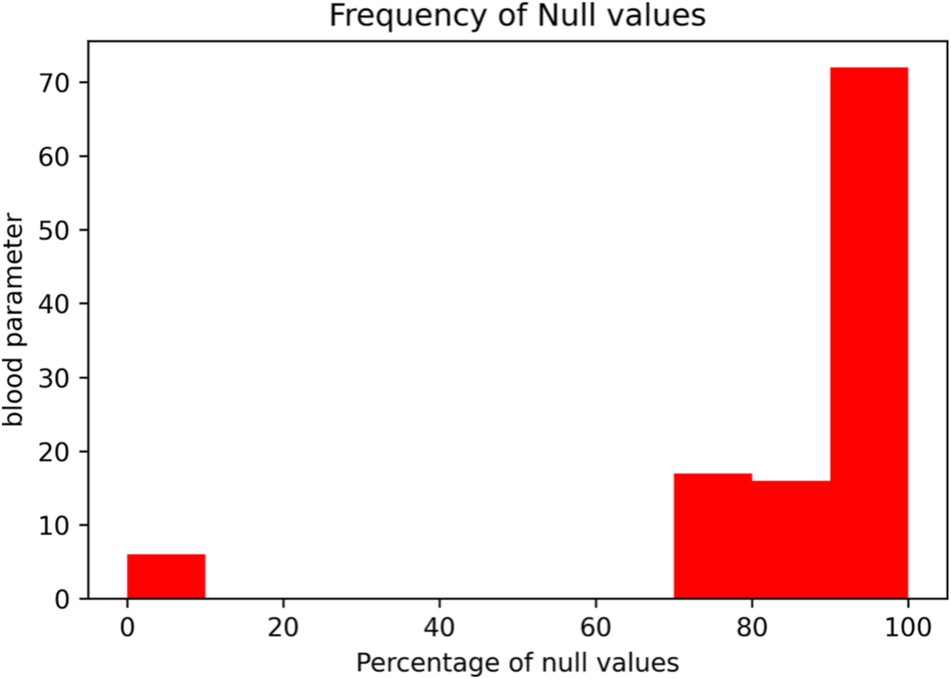
Null values present in attributes

Missing values imputation, elimination of outliers and balancing the data are the three major phases in data preparation. Our dataset contained a lot of null values and Fig. 2 shows that over 90% of the blood parameters had a lot of missing values.

Imputing those values with statistical parameters (mean, median, mode) would render the model useless. Therefore, all the columns that contained at least 90% of the null values were removed. After the removal, there were 39 attributes left. The parameter "parainfluenza 2" had only one value (variance = 0) and was dropped. 3596 rows of 5644 cases had null values above 80%. The dataset was trimmed further and all patient records were deleted that had more than 26 null values and all attributes and tests not related to COVID-19 were removed. The presence of antigens was mentioned in at least nineteen columns and the values were binary. These columns were used to confirm whether the patient tested positive for other viral infections such as Adenovirus, Parainfluenza, Metapneumovirus, etc. The results of these tests were combined together to form an attribute called “has-disease” and this attribute suggested if at least one of the respiratory infections were present. The dataset was already normalized except for a single column named "patient age quantile" and the values ranged from 1 to 19. The magnitude of the features can affect the results drastically in some AI algorithms. Hence, the age parameter was normalized in the range [−3,3] to prevent the impact of attributes with various scales.

After data pre-processing, 18 columns and 602 rows remained. The final set of features that were chosen are described in Table 2. The dataset contained 84 positive and 518 negative cases confirmed by RT-PCR tests. Thus, the dataset still had the problem of severe data imbalance (1:6 ratio). The proposed model uses the SMOTE technique that is available in the “imblearn” python library. This innovative technique balances the dataset by oversampling the minority-class instances.

**Table 2:** Feature description of the final selected parameters

Sl.no	Abbreviation	Feature	Description	References
1	AGE	Patient age quantile	Specifies the age of the individual	–
2	MPV	Mean platelet volume	Mean size of platelets presents in blood. It is known to increase in the presence of COVID-19	[74]
3	RBC	Red blood cells	The bone marrow produces fresh red blood cells. The red blood cell carries oxygen and removes carbon dioxide from the body	[75]
4	LYM	Lymphocytes	These are part of the person's immune system and are created by the lymph nodes and bone marrow. They tend to decrease for severe COVID-19 patients	[76]
5	MCHC	Mean Corpuscular haemoglobin concentration	Average quantity of haemoglobin present in each of the red blood cells	[77]
6	WBC	Leukocytes	They are also called white blood cells. They defend the body against various infections and threats. The count has increased in COVID-19 patients according to numerous studies	[78]
7	BAY	Basophils	They are a part of white blood cells	[79]
8	EOS	Eosinophils	They help in promoting inflammation that controls the infection. Eosinophil count is reduced for COVID-19 patients	[79]
9	MCV	Mean Corpuscular volume	Average volume of red blood cells. They increase or decrease depending on the average red cell size	[74]
10	MON	Monocytes	They are white blood cells that focus on healing and repair	[80]
11	PLT	Platelets	They form clots and prevent bleeding. COVID-19 patients often have mild thrombocytopenia	[81]
12	RBCDW	Red blood cell distribution width	The range of volume and size of red blood cells	[75]
13	-	Has_disease	A variable that has been created by combining all the other disease columns for this research. It specifies whether the patient suffers from other viral diseases	–

**Fig. 3:**
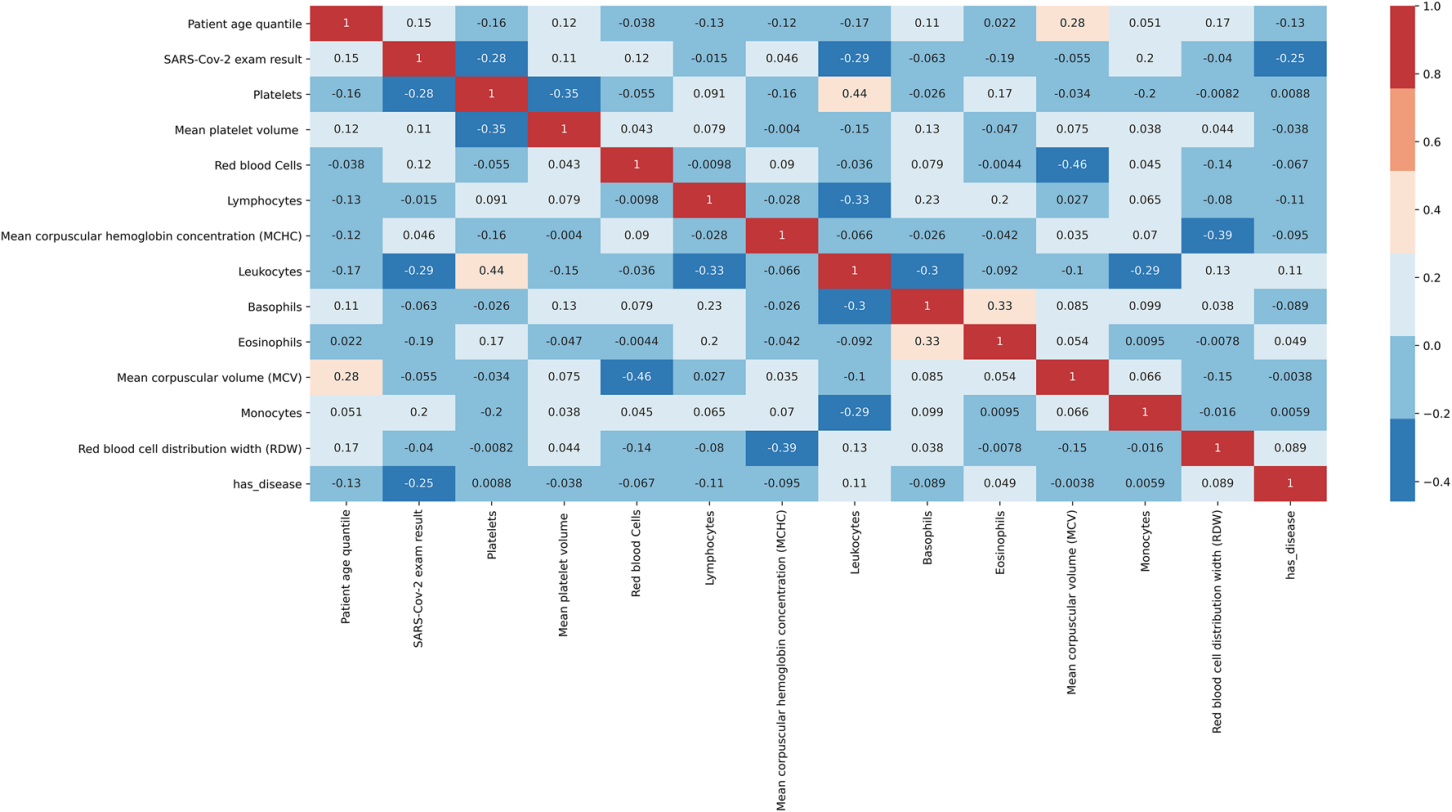
Pearson co-relation matrix

After completing the process of feature engineering, we proceed to feature selection. Pearson’s co-relation coefficient (PCC) was used to evaluate the co-relation between the attributes to remove the non-essential and redundant blood markers as shown in Fig. 3. Features that showed strong co-relations that indicated COVID-19 were also observed. It was seen that eosinophils, platelets, leukocytes and the has_disease attribute showed a negative co-relation (The values of these blood parameters decreased for COVID-19 patients), while monocytes, haemoglobin, red blood cells and age showed a slight positive co-relation (The values of these blood parameters increased for COVID-19 patients) as described in Table 3. Some characteristic parameters had a very high degree of interdependence. To reduce noise, we must remove parameters with a high degree of co-linearity between them. Haematocrit and haemoglobin had a co-relation factor of 0.97 between them and also had a strong positive relationship with red blood cells (0.87 and 0.84). We decided to retain red blood cells since they had the highest co-relationship with the target variable. MCV (Mean Corpuscular volume) and MCH (Mean Corpuscular Height) were the other two highly associated variables. MCV was retained since it was more co-related to the target label (−0.055 vs −0.028).

**Table 3:** Correlation coefficient and r value of the final blood parameters

Dependent features	Result Label	*r* value	Relationship co-relation
Age	RT-PCR test	0.15	Weak positive correlation
MPV	RT-PCR test	0.11	Weak positive correlation
RBC	RT-PCR test	0.12	Weak positive correlation
LYM	RT-PCR test	− 0.015	Very weak negative correlation
MCHC	RT-PCR test	0.046	Very weak positive correlation
WBC	RT-PCR test	− 0.29	Weak negative co-relation
BAY	RT-PCR test	− 0.063	Very weak negative correlation
EOS	RT-PCR test	− 0.19	Weak negative co-relation
MCV	RT-PCR test	− 0.055	Very weak negative correlation
MON	RT-PCR test	0.2	Weak positive correlation
PLT	RT-PCR test	− 0.28	Weak negative co-relation
RBCDW	RT-PCR test	− 0.04	Very weak negative correlation
Has_Disease	RT-PCR test	− 0.25	Weak negative co-relation

### Methodology

Since they are highly efficient with imbalanced data, xgboost, random forest and logistic regression were used as cutting-edge prediction models in this research. The KNN algorithm was also tested.

Random forest (RF) is a decision tree agglomeration approach that constructs many trees using a resampling procedure known as bagging (bootstrap aggregation) [82]. Resampling with replacement is used to create a huge number of decision trees. Every tree's node is divided using a subset of the tree's characteristics that are chosen at random. A simple unweighted majority vote is used to determine the most often predicted class for new data from the (aggregated) decision trees. When more trees are introduced, random forests do not overfit. Instead they provide a limiting value of the generalisation error, as indicated in Eq. 1. The number of trees used for classification was varied with the following values (10, 50, 100, 200, 500), split ratio of (1,2,4,8,16,24) and minimum leaf nodes of (1,2,5,10,15,30).

1 $$Px \, ,y \, (Pg(h\left( {X,0} \right) \, = \, Y) \, \mathop {{\text{max}} }\limits_{j = Y} Pg(h\left( { \, X \, , \, 0 \, } \right) \, = \, j) \, < \, 0)$$

By trying to compare an unlabelled data point to the training dataset, the K-nearest-neighbour (KNN) classifier improves considerably. It finds the K most related data-points, which are termed as KNNs [83]. A metric that measures distance such as Euclidean or Manhattan distance is widely utilized to determine proximity. This technique then assigns the given data point to the KNN's most familiar class. The number of potential nearest neighbours for KNN chosen were (2,3,5,8,10,12,15,20). XGBoost is an ensemble approach to build a series of trees successively [84]. A tree's performance is enhanced in each iteration based on the preceding iteration's results. The three components involved in any boosting algorithm are a loss function, an additive model and a weak learner (e.g., a decision tree). XGBoost used the same parameters as random forests with an additional learning rate parameter that was varied with the following values (0.01, 0.05,0.1). LR algorithm estimates the maximum probability of data-points pertaining to a particular label based on the values of the laboratory markers that are independent in nature [85, 86]. The model can then be used to make predictions that a data-point belongs to a particular label. The sigmoid function is commonly utilized to generate a logistic regression model. The data points are expected to follow a linear function. The following is a description of LR.

2 $${\text{log}}\left( {P(X} \right)/1 - P\left( X \right)) = \beta_{0} + \beta_{1} X$$

where *P* is the maximum probability that *X* is a member to class C and β_0_ and β_1_ are the parameters of the model. Testing was done using a ridge regression penalty of (11,12) and a sparsity of (100,10,1,0.1,0.01,0,001).

**Fig. 4:**
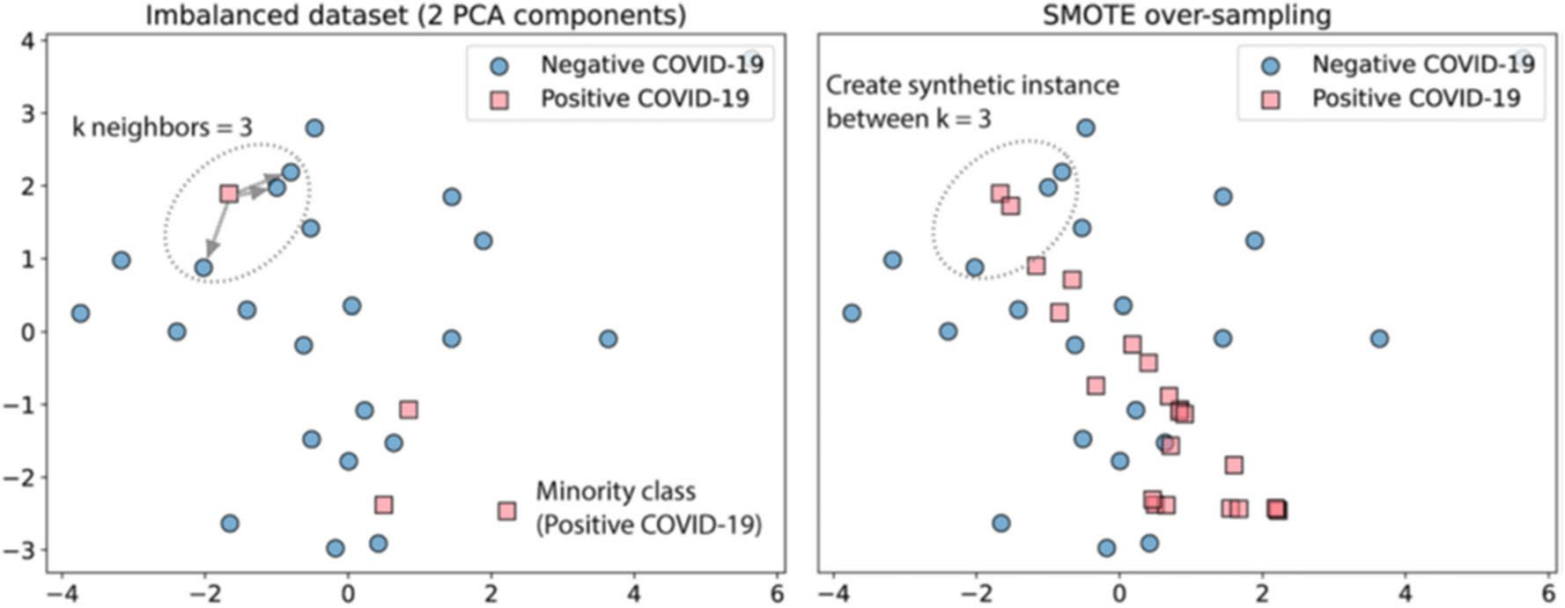
An example of synthetic sampling by SMOTE overall flow diagram is given below [34]

**Fig. 5:**
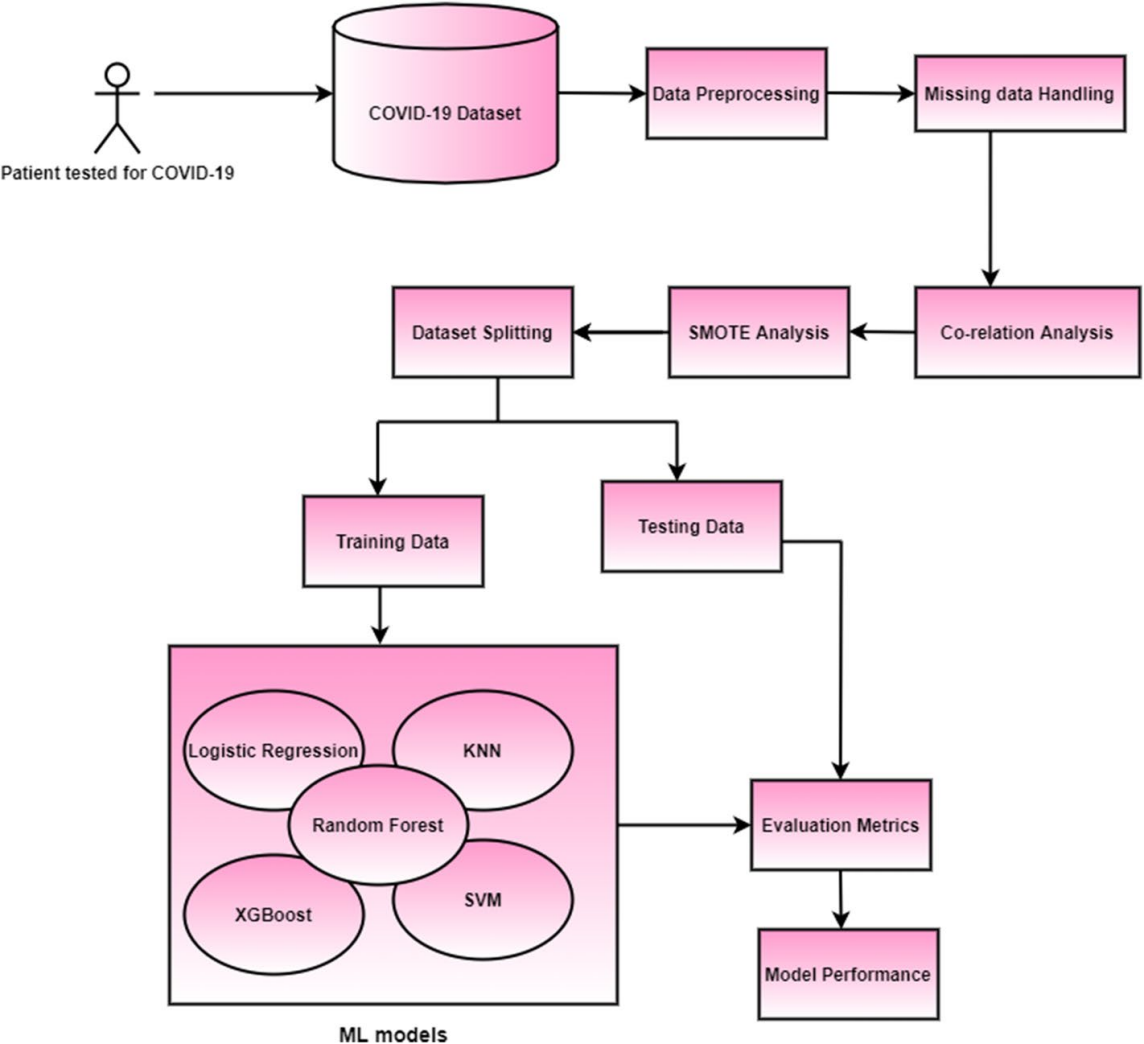
Block diagram describing the proposed method for the classification of COVID-19 based on blood sample data

The SMOTE [87] algorithm was then utilized to train every classifier. This approach synthetically oversamples minority-class data, producing the same occurrences in the training data for the positive and negative classes. As demonstrated in Fig. 4, this strategy resamples by producing an optimal synthetic sample from the k neighbours adjacent to the model. For this research, we used a set of *k* = 3 neighbours. We then selected the optimal of the five models produced for each classifier and retrained them in five separate iterations using their hyperparameters to assess their generalizability. We divided the dataset into 80 per cent for model training and 20 percent for model testing. With the imbalanced data in mind, we reran the SMOTE algorithm, but this time only for the training data, synthetically super sampling the minority-class data for each of the iterations. The overall proposed methodology is pictorially shown in Fig. 5.

## Results and Discussions

This section assesses the proposed machine-learning models and examines the outcomes. The first subsection discusses about the significance of various metrics used in our research, the second section compares the performances of various models using the above metrics. Feature importance using SHAP and random forest are examined in subsection three. Discussions on the important blood parameters that can diagnose COVID-19 are portrayed in the last subsection.

### Performance Metrics

Our prediction models are evaluated using a variety of performance indicators. The reliability of these models was assessed using the following performance indicators: AUC, accuracy, sensitivity, specificity, f1-score, and brier score. The confusion matrix is used to determine true positives’ (TP), false positives’ (FP), false negatives’ (FN) and true negatives’ (TN) as indicated in Table 4. The cases of TP occur when COVID-19 patients are correctly predicted and the cases of TN occur when COVID-19 negative patients are correctly predicted. The number of cases that are predicted incorrectly are determined by false positives and false negatives.

**Table 4:** Classification results

Model	Accuracy	Specificity	Sensitivity	F1-score	AUC	Brier score	Best parameters
Simple random forest	0.60	0.71	0.33	0.66	0.69	0.23	–
Random forest after feature selection	0.89	0.97	0.35	0.84	0.90	0.115	–
Random forest after hyper parameter tuning (Randomized searchCV)	0.88	0.96	0.41	0.84	0.92	0.115	{‘n_estimators’: 10, ‘min_samples_split’: 2, ‘min_samples_leaf’: 2, ‘max_features’: 10, ‘max_depth’: 128}
Optimal random forest (After SMOTE)	0.92	0.96	0.71	0.85	0.92	0.082	{‘n_estimators’: 500, ‘min_samples_split’: 4, ‘min_samples_leaf’: 1, ‘max_features’: ‘8’, ‘max_depth’: 32}
Optimal LR	0.85	0.87	0.70	0.81	0.89	0.157	{‘penalty’: ‘l2’, ‘C’: 100}
Optimal KNN	0.75	0.77	0.59	0.73	0.68	0.25	{‘weights’: ‘distance’, ‘p’: 1, ‘n_neighbors’: 2}
Optimal XGBoost	0.88	0.93	0.65	0.83	0.88	0.123	{‘n_estimators’: 100, ‘max_depth’: 8, ‘gamma’: 0, ‘colsample_bytree’: 0.8}

*Accuracy* The fraction of accurately predicted cases (Both COVID-19 negative and positive) in the entire data. The accuracy of a model in percentage is calculated using the following formula:

3 $${\text{Accuracy = }}\frac{{{\text{TP}} + {\text{TN}}}}{{{\text{TP}} + {\text{TN}} + {\text{FP}} + {\text{FN}}}}$$

*Specificity* It defines the percentage of genuine negatives correctly predicted by a model. In our research, the total proportion of patients who were not infected with COVID-19 and were correctly predicted as negative cases by the classifier. A classification model with good specificity has a high TN and less FP rates. The formula for calculating specificity is presented in Eq. (2):

4 $${\text{Specificity}} = \frac{{{\text{TN}}}}{{{\text{TN}} + {\text{FP}}}}$$

*Sensitivity (Recall)* It is the total percentage of true positives for the dataset. In our research, the percentage of actual coronavirus patients who were accurately identified as COVID-19 patients by the classifiers. A model with good sensitivity always has a high number of TP and less FN values. Equation (3) is used to calculate sensitivity.

5 $${\text{Sensitivity}} = \frac{{{\text{TP}}}}{{{\text{TP}} + {\text{FN}}}}$$

*F1-Score* It is a metric that measures model performance based on precision and sensitivity. It's used to assess binary classification algorithms that categorise examples as either "positive" or "negative." The F1-score is also the harmonic mean of the model's recall and precision. It is calculated using the formula:

6 $$F1 - {\text{score}} = 2 \times \frac{{\left( {{\text{Precision}} \times {\text{Recall}}} \right)}}{{{\text{Precision}} + {\text{Recall}}}}$$

*ROC Curve* The receiver operating characteristic (ROC) curve portrays the relationship between true positive rate (TPR) and the false positive rates (FPR). The area under curve (AUC) represents the area within the ROC curve and indicates how well the classifier distinguishes between its two categories. The higher the AUC, the better the predictions of the models.

*Brier Score* Brier score is a metric for assessing the quality of a probability score that has been forecasted. This is identical to the mean squared error. However, it only applies to prediction probability scores with values ranging from 0 to 1. A perfect accurate sample will have a brier score of 0 and 1 represents perfect inaccuracy. It is calculated using the formula:

7 $${\text{BS }} = \frac{1}{N} \mathop \sum \limits_{t = 1}^{N} \left( {f_{t} - O_{t} } \right)^{2}$$

where *N* = number of samples, *f*_*t*_ = forecast probability, *O*_*t*_ = is the actual outcome.

### Evaluation of Predictive Models

Early COVID-19 prediction can help decrease the significant load on health care facilities by assisting in the diagnosis of infected patients. In this research, RF, LR, KNN and XGBoost supervised classifiers were utilized for the prediction of the deadly virus. The performances of the above models are depicted in Table 5. Randomized search technique was utilized to obtain the optimal parameters for the evaluation of the classifiers. The SMOTE technique was then applied on each of the above five models. COVID-19 positive patients(minority-class) were synthetically over-sampled using this strategy. This resulted in more balance in the training data between the positive and negative classes. A total of *k* = 3 neighbours were chosen for this specific task. We built final models using the fivefold cross validation, which specified the number of external splits. As a result, we selected the perfect four models and retrained them in five iterations using the given hyperparameters to assess their generalizability. We split the data in half for each cycle, using 80% for training and the rest as testing.

**Table 5:** Normalized confusion matrices for test data set

(a) Random forest		Actual	
		Negative	Positive
Predicted	Negative	0.94	0.06
	Positive	0.41	0.59

(b) Logistic regression		Actual	
		Negative	Positive
Predicted	Negative	0.87	0.13
	Positive	0.30	0.70

(c) KNN		Actual	
		Negative	Positive
Predicted	Negative	0.70	0.30
	Positive	0.41	0.59

(d) XGBoost		Actual	
		Negative	Positive
Predicted	Negative	0.93	0.07
	Positive	0.47	0.53

For each actual class, the corresponding row sum is 1.0

**Fig. 6:**
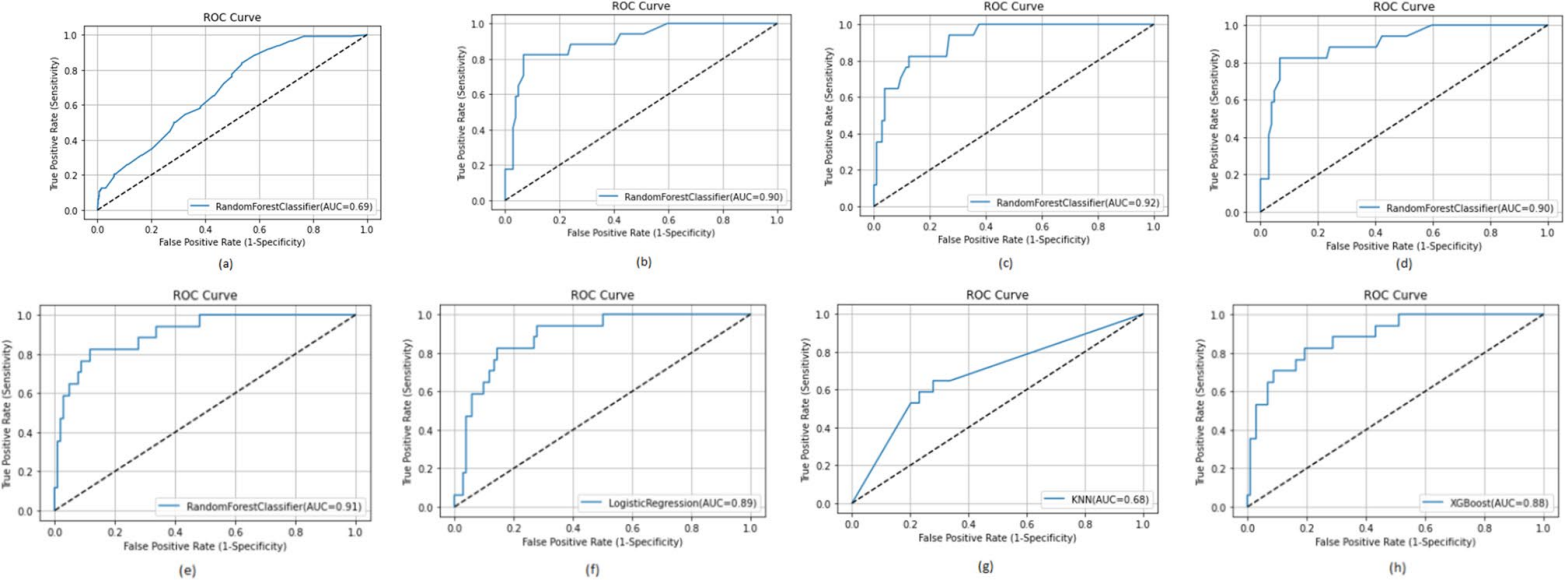
AUROC curves of the various ML algorithms as follows: **a** Initial RF model; **b** RF model after pre-processing; **c** RF model after hyperparameter tuning; **d** Model after SMOTE Analysis; **e** Optimized RF model; **f** Logistic Regression; **g** KNN; **h** XGBoost

In comparison to other models, the random forest model produced good results. After data pre-processing and SMOTE analysis, the best model had a 92% accuracy. The accuracy of KNN, logistic regression and XGBoost were 75%, 85% and 88% respectively. The percentage of COVID-19 positive patients properly predicted is revealed by sensitivity (recall). Our models, however, did not do well in terms of sensitivity. A maximum sensitivity of 71% was achieved using the random forest technique. Although the sensitivity attained was not particularly impressive, it was nevertheless adequate given the dataset's complexity. The number of COVID-19 negative patients correctly detected is calculated using specificity. Random forest obtained the highest specificity of 96%. KNN, LR and XGBoost obtained a specificity of 77%, 87% and 93% respectively. Specificity define the number of coronavirus negative patients identified correctly. A maximum specificity of 96% was obtained by the RF classifier. The specificity of the LR, KNN and XGBoost algorithms were 87%, 77% and 93% respectively. F1-score is a measure of recall and precision as suggested by Eq. 1 and it considers both false negative and false positive results. Random forest model obtained the maximum F1-score of 85%. LR, KNN and XGBoost obtained 81%, 73% and 83% respectively. The Receiver Operator Characteristic (ROC) curve is a binary classification evaluation metric as shown in Fig. 6. It is a probability curve that represents the true positive rate (sensitivity) against the false positive rate (1—specificity) at distinctive threshold values. These plots are created by changing the decision threshold and examining the TPR and FPR for each value. The better the model’s discrimination power in the diagnostic test, the closer the area is to 1. Random forest model obtained the optimal AUC of 91%. The AUC obtained by LR, KNN and XGBoost were 89%,68% and 88% respectively. Brier score is a metric for assessing the quality of a probability score that has been forecasted. This is identical to the mean squared error. However, it only applies to the prediction probability scores with the values ranging from 0 to 1. Random forest achieved the best brier score of 0.09. The LR, KNN and XGBoost obtained a brier score of 0.15, 0.25 and 0.123 respectively.

The best random forest model used 500 decision trees (n-estimators), the maximum number of attributes while splitting the node was eight (max_features), the minimum number of samples required for internal node split (min_samples_leaf) was 1. The maximum depth of each tree identified was 32 (max_depth). These features were identified after various iterations performed by the randomizedSearchCV algorithm. The logistic regression model was also able to deliver good results. Penalty and sparsity(c) are important parameters in a logistic regression model. Generally large values of c give more freedom to the classifier. For our model, 100 was the optimal sparsity value along with a regularization(penalty) of l2 (Ridge regression). Ridge regression forces the weights towards zero and adds “squared magnitude” of co efficient as penalty term to the loss function. This algorithm was similar to random forest, but was slightly less able to correctly classify COVID-19 positive patients.

The KNN algorithm achieved average results. The number of nearest neighbours (*k*) were varied and the optimal value of k was 2. The distance was calculated using the Minkowski distance (*p* = 1). This distance is equivalent to Manhattan distance (If p was 2, Euclidean distance would have been chosen). The XGBoost classifier also achieved excellent results. The best XGBoost model used 100 decision trees(n-estimators). The maximum depth of each tree identified was eight. This boosting algorithm also uses a unique regularization parameter called “gamma”. Unlike the parameters “max_depth” and “min_child_weight” that evaluates using “within tree” information, gamma uses “across trees”. Hence, nodes are added only if the gain associated is large than the gamma value.

Coronavirus can be predicted using ML models as a retrospective evaluation procedure. This research identifies how ML infection models can be constructed, confirmed and utilised to quickly identify COVID-19 cases. The research also highlights the crucial significance of ML classifiers in the diagnosis and prevention of COVID-19. This helps in reducing the significant load on front line health workers and also in poor countries that suffer from lack of technology and healthcare resources.

### Feature Importance

**Fig. 7:**
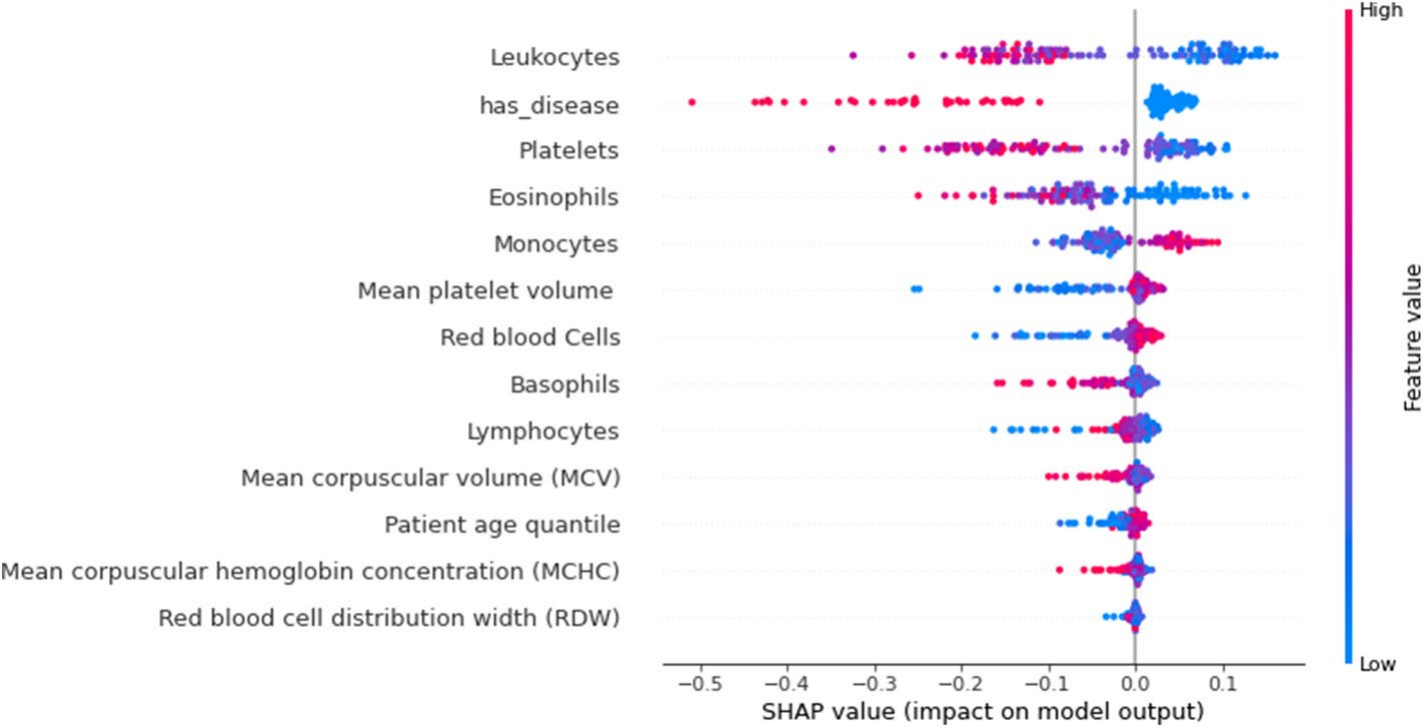
Feature importance using SHAP

Glit-edge clinical judgments made using machine-learning models in healthcare settings will have an impact on patients' lives regardless of numerous legal and ethical implications. As a result, diagnostic models that are both interpretable and precise are in great demand [88], 89. Model interpretability in the medical field refers to the ability of healthcare practitioners to comprehend how the algorithm utilizes input information to make predictions and to check the classifier’s outputs before taking decisions and to defend treatment decisions based on the ML models [90]. As a result, feature relevance estimations based on causality are crucial for predictive model interpretability and robustness. The Shapley Additive exPlanations (SHAP) technique [91] and random forest algorithm were used to analyse each feature's value in deciding the anticipated prediction to comprehend the suggested AI models. SHAP examines a model using Shapley values that describes how each attribute contributes to the COVID-19 prediction. Figure 7 shows a density scatter plot that reveals shapley values and combines feature relevance with effect of various features in both Sars-CoV-2 positive and negative patients. On the left, features are arranged in order of their significance. The colour on the right defines the feature value, the colour blue signifies a lower value and red signifies a higher value. Low value of leukocytes contributes the highest to the prediction model in diagnosing coronavirus positive cases, as seen in Fig. 7. Low value for eosinophils and platelets found using laboratory results in positive individuals in clinical settings are also important for our predictive model. The presence of other diseases also indicates COVID-19 negativity. In addition to this, the dots on the chart are coloured according to the normalised values of the patient's blood markers, such as the number of leukocytes. The value of a trait decreases as it gets closer to blue, and increases as it gets closer to pink. As a result, a low value of eosinophils, as well as the platelet count, as seen in blue, has a beneficial influence on the COVID–19 output.

**Fig. 8:**
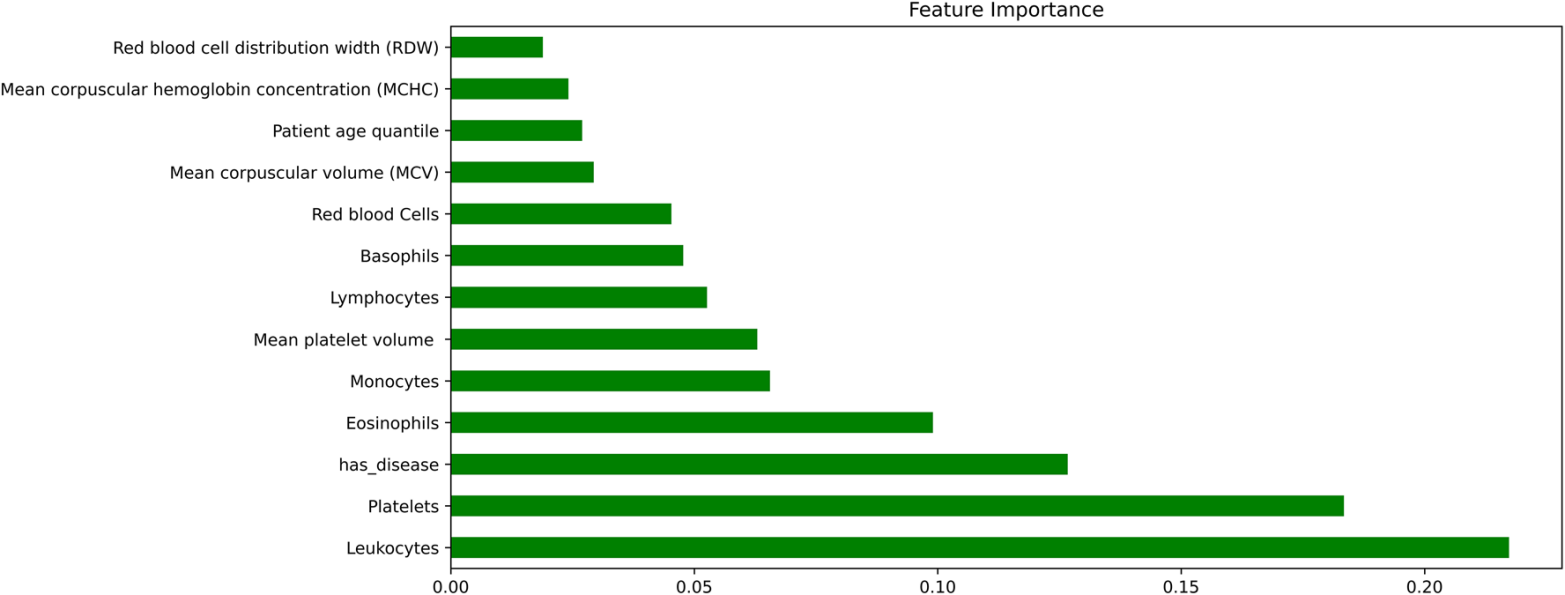
Feature importance using random forest

**Fig. 9:**
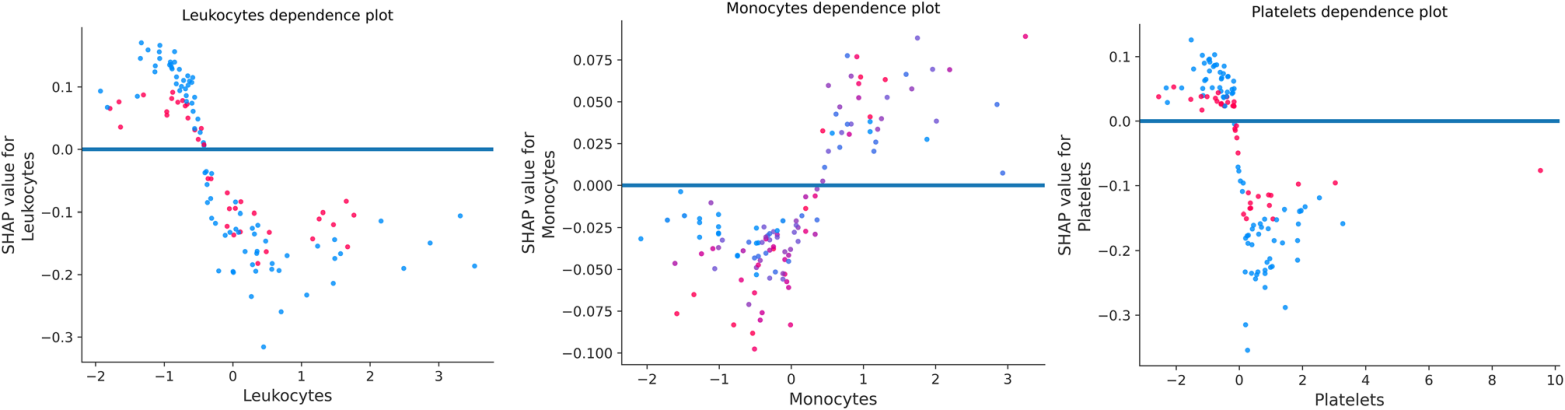
Marginal effect of Leukocytes, Monocytes and Platelets on COVID-19 outcome

In random forest, each predictor feature is randomly shuffled and these methods determine the importance of each attribute by monitoring the impact on model accuracy. The value of features is shown in Fig. 8 using random forest. It confirms and validates the most important features obtained from the SHAP Analysis. Figure 9 illustrates the marginal effect plot of blood markers on the target output that may be used to visualise the distribution of RT-PCR results across the sample. There is a central trend around normalised values for leukocytes and platelet levels, which are the lowest of these variables. This is in line with other researches, which suggests that platelet count can represent pathological alterations in COVID–19 cases [51]. This pattern is also observed in monocytes and appears to be linked to illness severity [52].

### Discussion on the Obtained Results

We identified a set of routine blood test values strongly linked with SARS-CoV-2 positivity in the retrospective research of coronavirus patients along with other patients with same symptoms but verified as COVID-19 negative. These characteristics may aid doctors in identifying potential infected patients before formal diagnostic test results are available.

It has been discussed by researches that the leukocyte count tends to decrease for COVID-19 patients [78]. Our research agrees with the same and leukopenia generally occurs with lymphopenia, even when there is a normal white blood count and this condition was also associated with disease severity. The part of eosinopenia in the diagnosis of COVID-19 was discussed in many researches. Eosinophil levels were lower in patients infected with coronavirus and was also associated with patient prognosis and mortality [79]. It also has a link to coagulation disorder biomarkers as well as tissue disorder biomarkers in the kidney, liver and other tissues. Thrombocytopenia is also a common observation in COVID-19 patients. According to many studies, COVID-19 may trigger platelet destruction [81]. Although the cause is uncertain, it has been linked to platelet membrane components in circulating immune complexes as well as anti-platelet member GPIIa49-66 Igh antibodies. Since the current COVID-19 outbreak, multiple investigations have provided a co-relation between the infection and lymphopenia, a condition marked by abnormally low lymphocyte levels. However, it is more common in the elderly, who have a greater death risk, especially in severe cases. Lymphopenia and elevated levels of specific cytokines such as IL-6, have been linked to this devastating disease in general [76]. Monocytes are innate immune cells that participate in inflammatory reactions, phagocytosis, antigen presentation and a range of other immune function process. In our research, monocytes count increase for COVID-19 patients and agree with other conducted researches [80].

The extraordinary health catastrophe caused by the pandemic has prompted several groups of researches to create AI applications with the goal of automation in COVID-19 diagnosis and screening. Despite this, only a few AI models have been designed which is solely based on routine blood tests. Formica et al. [92] designed an AI model based on clinical and laboratory parameters with 83% sensitivity and 82% specificity. However, only a small sample was included (171). Banerjee et al. [65] used AI models on a public dataset containing 600 cases (39 positive COVID-19 patients). They found high specificity (91%) but extremely low sensitivity (43%) rendering it inappropriate for early detection. Avila et al. [93] designed a model that used a Bayesian approach with 76.7% sensitivity and specificity using the same dataset as [65]. Joshi et al. [94] used a CBC dataset for training a logistic regression model on a dataset that achieved higher sensitivity (93%) and lower specificity (63%). Finally, Yang et al., [45] in a recent study, constructed a gradient boosting model using 27 parameters (42% were COVID-19) including both blood count and biochemical analysis. An AUC of 0.85 was reported. The summary of comparisons is given in Table 6.

**Table 6:** Comparison between the related studies and the proposed work

Reference	Accuracy of best model	Sensitivity of best model	Specificity of best model	AUC of best model	ML models used
[92]	–	83%	82%	–	Only Statistical analysis
[65]	85%	91%	43%	80%	ANN, RF, glmnet
[93]	–	76%	76%	84%	Naïve Bayes
[94]	–	93%	63%	95%	Many models
[45]	–	–	–	85%	XGBoost
Proposed	91%	94%	71%	91%	RF, XGBoost, LR, KNN, SVM

To overcome the constraints of the previous models, we used machine learning to analyse the result of routine blood examinations, which are typically available for inpatients in lesser time interval and at a cheaper cost than molecular and radiographic tests. For the dataset, we used four models that are commonly deployed and adapted in medical ML. There are numerous benefits of utilizing electronic medical records, including patient information availability and security, data integration/standardization and procedural automation. Coronavirus is known to be highly contagious and quick assays to diagnose the disease are currently available. As a result, we underline that the proposed approach aimed at assisting physicians in their decision-making by offering more information. Furthermore, a significant difference of the proposed procedure is the display of model explainability, making the resources understandable to the medical personnel and thus assisting them in the final diagnosis.

## Key Issues and Future Directions

This section discusses about the various challenges and the clear directions for future researches.

### Key Issues

- *Diagnosing COVID-19 from other viral infections* Blood parameters such as eosinophils, platelets, leukocytes and monocytes and others vary for other viral infections too. Extensive research is required to find the parameters that can be used to distinguish coronavirus from other viral diseases. Other tests might be required to confirm the highly infectious virus.
- *Single centric data* The models lacked from external validation since the data belonged to a single hospital. It is very important to consider data from different geographical territories to validate the effectiveness of the models.
- *Data Imbalance* The data obtained is extremely imbalanced. The number of COVID-19 cases are extremely few compared to the non-COVID-19 cases. For any ML algorithm, it is very important for the data to be balanced, since balanced datasets are known to give good performance.
- *Data Consistency* The original values of the blood parameters are not known since the dataset was already normalized (z-normalization) by the hospital. It is extremely important to know the exact values for various statistical analysis.
- *Lack of availability of important markers* From various researches, it has been proved that various markers such as CRP, D-Dimer, LDH and ferritin are very important in diagnosing and predicting the severity of coronavirus. However, the results of those tests were not available in the dataset.

### Future Directions

- *Getting a better dataset* For subsequent researches, we aim to collect a more balanced dataset. Various important blood parameters (D-dimer, LDH, CRP) should also be included. The severity of COVID-19 could also be predicted.
- *Usage of Multimodal ML algorithms* Ensemble algorithms are a combination of more than one base ML model that is used to improve the accuracy. Rather than creating a single model, ensemble methods consider a large number of models and combines them to produce a single final reliable classifier.
- *Deep learning* Unlike ML, deep learning models can perform feature engineering without external intervention. Using GPU’s and TPU’s will also enable a faster and efficient learning. The neural network models also work efficiently with unstructured data.
- *Medical Validation* After validation of the ML models by clinical experts, the models can be deployed in various health care facilities in the near future to reduce the burden on health care workers.
- *Combining multiple diagnostic methods to improve accuracy* These models can be used with other AI deployed models that use CT- Scans and X-ray data to improve the model performance. Integration of these models has the potential to yield optimal results.

## Conclusion

COVID-19 must be identified early for patients to receive appropriate treatments and prevent the pandemic from spreading. Recent research has revealed the use of laboratory testing for preliminary patient screening, which is supported by the factuality that clinical exams are relatively less expensive, expensive and readily accessible in most treatment centres. We initially conducted an overview of current SARS-CoV-2 detection strategies utilising regular laboratory and clinical data in this article to encourage researchers to develop efficient prediction models to tackle this infectious disease. Later, multiple ML models for diagnosing COVID-19 using several clinical and laboratory markers were developed. Since four separate classifiers were utilised, structural diversity was achieved. By comparing the positive outcomes and results to the previous researches, the classifiers' effectiveness and reliability for diagnosis were also established. We used the SHAP method to assess the value of each attribute in impacting the expected result to comprehend the suggested findings better.

However, some issues must be overcome in order for ML to advance in accurate and automated COVID-19 diagnosis, especially in professional healthcare settings. High quality datasets, external validation and rigorous testing with the guidance from various doctors and healthcare personnel must be performed in the future.
